# Successful Pregnancy after Simultaneous Pancreas-Kidney Transplantation

**DOI:** 10.1155/2011/983592

**Published:** 2011-07-10

**Authors:** A. Smyth, G. Gaffney, D. Hickey, D. Lappin, D. Reddan, F. Dunne

**Affiliations:** ^1^Department of Endocrinology, Galway University Hospitals, Galway, Ireland; ^2^Department of Obstetrics & Gynaecology, Galway University Hospitals, Galway, Ireland; ^3^Department of Urology & Transplantation, Beaumont Hospital, Dublin, Ireland; ^4^Department of Nephrology, Galway University Hospitals, Galway, Ireland; ^5^School of Medicine, National University of Ireland, Galway, Ireland

## Abstract

The effect of pregnancy on simultaneous pancreas-kidney transplant recipients has previously been described, but experience is limited. We describe the case of a thirty-five-year-old female who previously underwent simultaneous pancreas-kidney transplant for type 1 diabetes mellitus-complicated nephropathy. An integrated multidisciplinary team including the transplant team, nephrologist, endocrinologist, and obstetrician closely followed progress during pregnancy. Blood glucose levels and HbA1c remained within normal limits, and she did not require insulin treatment at any point. She experienced deterioration in renal indices and underwent an uncomplicated, elective Caesarean section at thirty-week gestation. She delivered a male infant of 1.18 kg, appropriate for gestational age, who had hypothermia and respiratory distress, which required intubation and ventilation and an eleven-week stay in the special care baby unit. At eighteen-month followup the infant shows normal development, and there has been no deterioration in either grafts' function.

## 1. Introduction

The first pregnancy in the recipient of a simultaneous pancreas-kidney transplant (SPKT) was reported in 1986 [1]. Increasing numbers of women of childbearing age worldwide are undergoing transplantation, including SPKT. The majority of data from pregnancy in transplant recipients comes from the renal transplant population. In SPKT recipients, the presence of two organs within the pelvis may pose additional risks. There are risks to both mother and newborn [2, 3] including miscarriage, preterm birth, foetal malformations [4], deterioration in maternal renal function, pre-eclampsia, infection, and pancreas-graft pancreatitis induced by normal delivery [5].

Diabetes is the leading cause of renal failure in adult patients, and in select cases, pancreas transplantation has been performed since the mid-1980s [6]. Pancreas transplantation has a beneficial effect on nephropathy [7], autonomic neuropathy [8], and peripheral neuropathy [9] which may lead to improved quality of life [10]. Renal impairment, such as that occurring secondary to diabetic nephropathy, may result in gonadal insufficiency with resultant infertility. SPKT can restore fertility in patients with diabetes and end-stage renal disease [11], and as such, successful pregnancy outcomes can be achieved [12, 13].

Compared to kidney transplant recipients, SPKT recipients have similar rates of spontaneous miscarriage and therapeutic abortion, but higher rates of preterm delivery, low birth weight, hypertension, infection, pre-eclampsia, acute rejection, and graft loss in later years [14]. Risks are reduced if pregnancy is planned and if the graft is functional at least one-year after transplantation without evidence of rejection; with normal blood pressure and stable immunosuppression doses [15]. We present a case of a planned pregnancy in such a patient.

## 2. Case Description

A thirty-five-year-old female with a complex medical history presented at six weeks of gestation. She had a history of type 1 diabetes mellitus diagnosed at the age of ten, complicated by retinopathy, bilateral cataract surgery, and nephropathy (which required renal replacement therapy). Six years previously, she underwent SPKT with the pancreas allograft sited in the right iliac fossa and the renal allograft in the left iliac fossa. The donor was CMV positive, and our patient was negative. Human leucocyte antigen (HLA) mismatching showed no HLA-A mismatch, one HLA-B mismatch, and two HLA-DR mismatches. Induction immunosuppressive therapy for transplant included antithymocyte globulin and in the postoperative state tacrolimus, mycophenolate mofetil, and prednisolone were prescribed in addition to standard antiviral (ganciclovir) and antifungal (fluconazole) therapies. Recovery from transplant was uneventful, and she soon achieved normoglycaemia and good renal allograft function, with serum creatinine of 92 mmol/L on the eighth postoperative day.

Due to the high-risk nature of these cases, pregnancy was planned, and during preconceptual counseling, treatments were optimized. Due to the risk of foetal teratogenicity, mycophenolate mofetil was changed to azathioprine. Further immunosuppressive doses were stable prior to pregnancy. There was evidence of good function of both renal and pancreas allografts with serum creatinine of 146 mmol/L with fasting blood glucose of 4.1 mmol/L. As she had a history of polycystic ovarian syndrome with prolonged amenorrhoea, she attended fertility services and required hormonal support to achieve pregnancy. Recombinant FSH was used to stimulate ovulation, and singleton pregnancy was achieved on the sixth cycle of treatment. Progesterone gel (8%) was used until week fourteen of gestation. Throughout the pregnancy, the transplant team as well as her nephrologist, endocrinologist, and obstetrician with a special interest in high-risk pregnancy closely followed her progress.

Despite normotensive prepregnancy, at nine weeks of gestation, she had high systolic and diastolic blood pressure readings on home monitoring, confirmed on clinical assessment. She was diagnosed with pregnancy-induced hypertension and commenced labetalol therapy, which was uptitrated throughout pregnancy. Vaginal bleeding occurred transiently at eleven weeks of gestation, which resolved spontaneously. Multiple scans for fetal well-being were performed, and no fetal anomalies were noted including normal amniotic fluid and placental structure and function. Ophthalmology reviews during pregnancy noted no deterioration in diabetic retinopathy. Tacrolimus doses were adjusted based on close monitoring of trough levels throughout the pregnancy, whilst prednisolone and azathioprine doses remained stable. Tacrolimus levels were stable throughout pregnancy and within the accepted target range of 6–10. Blood glucose monitoring, fasting blood glucose, and serial glycosylated haemoglobin (HbA1c) levels were in the normal range throughout pregnancy. Insulin treatment was not required at any stage before, during, or after pregnancy.

Serum creatinine was stable prior to pregnancy but steadily increased throughout the pregnancy. She experienced deterioration in renal function at twenty-six weeks of gestation and was admitted for monitoring. Intramuscular betamethasone to enhance foetal lung maturity was administered during this admission. Renal indices were transiently stabilized, but due to further deterioration of delivery was planned for thirty weeks and three days of gestation. She underwent elective Caesarian section (Pfannenstiel incision) under epidural anaesthesia, performed by her local obstetrician with assistance from the original transplant surgeon to monitor both grafts. The procedure was uncomplicated, and no deterioration in graft function was observed.

She delivered a male infant who had APGAR scores of seven and nine at five and ten minutes, respectively. Birth weight was 1.18 kg, which was appropriate for gestational age based on foetal birth weight centile. He was hypothermic and had evidence of respiratory distress, which required intubation and ventilation, and he was admitted to the special care baby unit, where he remained for eleven weeks. Following discharge to home, the infant experienced no further complications. Following delivery, our patient experienced progression of hypertension transiently and required the addition of amlodipine for three days. Following this, she developed labile blood pressure, and all antihypertensive agents were discontinued. At eighteen-month followup, she had no evidence of hypertension.

## 3. Discussion

Multiple pregnancies have been reported in SPKT recipients, including those who required assisted reproductive technology [16], such as in our case. Overall, SPKTs are felt to be able to provide sufficient metabolic support throughout a pregnancy [17]. These pregnancies are felt to be of high risk and require close monitoring with integrated multidisciplinary care from healthcare professionals [18, 19] including nephrology, obstetrics, endocrinology, and paediatrics [20]. As the risk of graft loss or adverse pregnancy outcome is highest in the first year after transplantation [15], patients should be counseled on the risks of graft loss, maternal morbidity and maternal mortality. The importance of contraception, following transplantation, should be stressed in order to facilitate planning of pregnancy [11].

A balance between the risk of graft rejection and the potential teratogenic effect of immunosuppressive medications must been struck. Importantly, inadequate levels of immunosuppression may result in acute allograft rejection [2]. Cyclosporine was traditionally the agent of choice, but newer agents such as tacrolimus are now frequently used, as was in our case. Tacrolimus-based regimens continue to carry a risk of prematurity, preterm delivery, low birth weight, and IUGR similar to other immunosuppressive agents [21] but overall are felt safe for use during pregnancy [22, 23]. Mycophenolate mofetil is absolutely contraindicated during pregnancy due to a high incidence of structural malformations [24]. It was for this reason that our patient was transitioned to azathioprine, which has been shown to be nonteratogenic [25, 26].

As pregnancy may affect the hepatic cytochrome P450 system with resultant fluctuations in serum tacrolimus level [27], which may result in the increase of tacrolimus requirement in pregnancy [28], frequent monitoring of trough levels was performed during pregnancy with appropriate adjustment of dosage. Tacrolimus toxicity could have contributed to elevated serum creatinine levels and hypertension [29], but this was not seen at any stage during this case. In the postpartum period, drug levels may increase dramatically, and continued close monitoring is advised. During followup, tacrolimus requirement rapidly decreased in our case, and with close monitoring, she did not develop any evidence of tacrolimus toxicity. Most reported cases of SPKT remain normoglycaemic during pregnancy, and gestational diabetes has been previously reported, particularly in those on both tacrolimus and prednisolone [30]. In this case, pancreatic function was normal before pregnancy, during pregnancy, and in the postpartum period. Although anatomically allografts are not likely to be damaged by foetal descent and vaginal delivery, the majority of deliveries in SPKT recipients are by caesarean section [31]. In our case, a combined decision was made for planned delivery by caesarean section with an experienced transplant surgeon available in the event of any complication, as suggested in the international guidelines [32].

This case highlights the difficulties encountered in the management of pregnancy in SPKT recipients including the need for planning of pregnancy, ensuring that immunosuppression doses are stable, and monitoring graft function and the importance of planning delivery. Despite multiple issues encountered in the management of this pregnancy, it was successful with overall good maternal and foetal outcomes, despite preterm delivery. In order to achieve successful pregnancy outcomes, we suggest that an integrated, multidisciplinary team including endocrinology, nephrology, obstetrics, and transplant surgeons jointly manages such cases.
